# Feasibility Study of a New Cherenkov Detector for Improving Volcano Muography

**DOI:** 10.3390/s19051183

**Published:** 2019-03-08

**Authors:** Domenico Lo Presti, Giuseppe Gallo, Danilo L. Bonanno, Daniele G. Bongiovanni, Fabio Longhitano, Santo Reito

**Affiliations:** 1 Department of Physics and Astronomy “E. Maiorana”, University of Catania, Via S. Sofia 64, 95123 Catania, Italy; domenico.lopresti@ct.infn.it; 2 National Institute for Nuclear Physics (INFN), Sezione di Catania, Via S. Sofia 64, 95123 Catania, Italy; danilo.bonanno@ct.infn.it (D.L.B.); fabio.longhitano@ct.infn.it (F.L.); santo.reito@ct.infn.it (S.R.); 3 National Institute for Nuclear Physics (INFN), Laboratori Nazionali del Sud, Via S. Sofia 62, 95123 Catania, Italy; danielebongiovanni@hotmail.it

**Keywords:** muography, Cherenkov radiation, Monte Carlo, Geant4, MATLAB, particle detectors

## Abstract

Muography is an expanding technique for internal structure investigation of large volume object, such as pyramids, volcanoes and also underground cavities. It is based on the attenuation of muon flux through the target in a way similar to the attenuation of X-ray flux through the human body for standard radiography. Muon imaging have to face with high background level, especially compared with the tiny near horizontal muon flux. In this paper the authors propose an innovative technique based on the measurement of Cherenkov radiation by Silicon photo-multipliers arrays to be integrated in a standard telescope for muography applications. Its feasibility study was accomplished by means of Geant4 simulations for the measurement of the directionality of cosmic-ray muons. This technique could be particularly useful for the suppression of background noise due to back-scattered particles whose incoming direction is likely to be wrongly reconstructed. The results obtained during the validation study of the technique principle confirm the ability to distinguish the arrival direction of muons with an efficiency higher than 98% above 1 GeV. In addition, a preliminary study on the tracking performance of the presented technique was introduced.

## 1. Introduction

Muon radiography—or briefly muography—is a promising technique which aims at resolving the internal structure of large size objects by taking advantages of the high penetrating power of cosmogenic muons. Although the properties of muons interaction with matter have been known for a long time, the investigation of their potential as a probe to give information of large structures is a recent development. The first attempts to produce muographic images to inspect large volumes date back to the middle of XXth century with the pioneer works of George [1] and Alvarez [2]. In last years it is possible to found an increasing number of papers discussing the application of muography to target as volcanoes, underground cavities, glaciers and also pyramids with impressive results [3,4,5,6,7,8,9].

The muography technique is based on the reconstruction of the incident direction of the detected muons after crossing the target object; for this purpose, a muography experiment requires a tracker detector with at least two position sensitive planes in order to reconstruct the particle trajectories. In general, the detector is able to track particles which come from two sides, front and back, and their incoming direction could be distinguished from the slope of reconstructed trajectories, assuming that the muon flux is downward oriented only. In this circumstance, muons detected, after being scattered near the detector and crossing it with an upward going direction, will be wrongly reconstructed [10]. This contamination, generally referred as “backward-scattered muons”, together with the other sources of background (hadronic and soft components [11,12] and forward-scattered muons [13]), produces an overestimation of the muon counts that could be critical when compared with a very tiny expected flux.

A proposed solution to overcome the background due in particular to backward-scattered particles, is the time-of-flight (TOF) measurement of the muons in traversing the detector [14]. The muon incoming direction can be distinguished by the difference between the detection time Δt in the external tracking planes of the detector. The path from starting to stopping points of time measure depends on how much the trajectory is inclined respect to telescope axis. Then, the time distribution will be characterized by two lobes for Δt>0 and Δt<0, respectively, with a superimposition for Δt≃0, corresponding to particles perpendicular to the detection planes, which is determined by time measurement resolution and detector size. Hence, an uncertainty remains about the incoming direction of fast muons, in particular for those with trajectories almost perpendicular respect to the tracking planes. This is clearly visible in ref. [10] which shows the TOF distribution for a data set obtained with a telescope whose outer matrices distance was equal to 60 cm. The ambiguity for Δt of near horizontal tracks, could be reduced by a substantial expansion of the distance for time measurement, at the expense of the portability and compactness of the detector. Furthermore, for a muon telescope based on scintillator strips with Wavelength Shifting fibers (WLS) readout, the propagation time of light through the fiber and its real path constitute other source of uncertainty.

In this paper a complementary solution for this problem, based on the directionality of Cherenkov emission, is investigated by means of Monte Carlo simulations. The results, reported in the following, show that muons incoming direction can be distinguished with an efficiency higher than 98% for muons with kinetic energy above 1 GeV for the best simulation scenario. Meanwhile, the possibility to develop a stand-alone muon telescope based on Cherenkov emission was considered and a preliminary study of the tracking performance of this detector is reported, showing promising results also for other charged particle tracking applications.

In the Results section the detector design and the working principle of the new technique are described, before reporting the summary of feasibility study data. First, the validation of the Cherenkov-tag detector for discrimination of particle incoming direction is discussed and, then, a first study of its tracking performance is introduced. In the Discussion section the results just exposed are examined in detail and the positive conclusion of the study conducted is argued. The Methods section is mainly devoted to the details of Monte Carlo simulations, performed by means of Geant4 toolkit, and to the statistical approach for SiPM behavior reproduction in MATLAB. In the Conclusion, after a brief outline the results already discussed, the future outlooks of this work is presented.

## 2. Results

**Figure 1:**
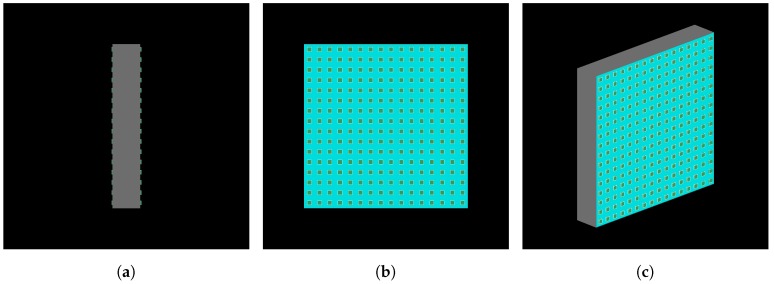
Lateral (**a**), front (**b**) and perspective visualizations (**c**) of the detector simulated in Geant4 for the configuration with a 16×16 array of SiPMs ($6\times 6\;{\mathrm{mm}}^{2}$ sized) for the optical readout of each sensitive face. The size of each radiator is $20\times 240\times 240\;{\mathrm{mm}}^{3}.$

The innovative Cherenkov-tag detector was designed as a possible upgrade of the muon telescope already working, developed inside the Muography of Etna Volcano (MEV) project [15]. It consists of two radiators, i.e., two plate of transparent material in which the emission of Cherenkov light takes place simultaneously to the passage of a charged particle with sufficient energy. The two radiator have a face in common, divided only by a light absorbing coating foil in order to prevent that the Cherenkov radiation generated in the first radiator traversed is reflected back or escapes from it and enters into the other plate. The opposite side of each plate is instrumented with light sensors to detect Cherenkov light, while lateral faces are also coated with light absorbing foils. The design of the detector is shown in Figure 1. This is a module composed of two radiator of transparent material with size $20\times 240\times 240\;{\mathrm{mm}}^{3}$. In sight of a possible upgrade for the MEV project telescope, with a sensitive area of 1 $m^{2}$, the final Cherenkov tag detector will be composed of a square array of 16 single modules to cover the sensitive area.

The working principle is quite simple: the Cherenkov radiation should be revealed only in the second radiator traversed by the particle, in which the light is emitted toward the instrumented face of the plate.

The Cherenkov threshold is usually given in terms of the ratio between particle velocity and the speed of light, $\beta_{th}=1/n$, where *n* is the refractive index of the medium. Since $E=\gamma m_{0}c^{2}$ and $\gamma =\left(1-\beta^{2}\right)$, the threshold can be expressed as:

(1) $$E_{th}=\frac{m_{0}c^{2}}{\sqrt{1-\frac{1}{n^{2}}}},$$

in which is evident the dependence on particle rest mass $m_{0}$. The mechanism of Cherenkov effect confines the photons to a cone with its vertex coincident with the point of first light emission. Also the aperture angle $\theta_{C}$ of the light cone is related to the particle velocity and to the refractive index of the medium according to the equation

(2) $$\cos\theta_{C}=\frac{1}{n\beta }.$$

**Figure 2:**
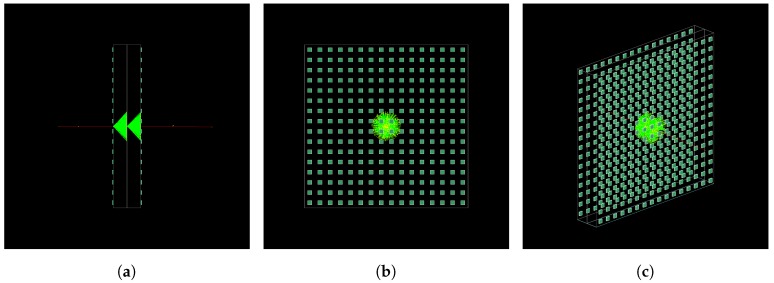
Lateral (**a**), front (**b**) and perspective visualizations (**c**) of event of a muon with $10^{5}\;\mathrm{MeV}$ kinetic energy, simulated in Geant4 for the configuration shown in Figure 1. The optical photons internally reflected at the interface between Plexiglas and air were suppressed to simplify the scene. Respect to Figure 1, Plexiglas and light absorbing foils are drawn only as wire-frame in order to visualize photons trajectories inside.

With this in mind, it is possible to imagine what happens when a charged particle enters the detector from an instrumented side. The emission of Cherenkov photons immediately begins if the particle energy is greater than $E_{th}$ and the photons produced in the first radiator will be directed toward the light absorbing foil, stopping in it. Then, the particle enters the second radiator and now the photons will be emitted in the direction of the light sensitive surface. An example of the described process is shown in Figure 2: the charged particle (red track) which produces Cherenkov emission is a muon with kinetic energy equal to $10^{5}\;Me\;V$ at the starting point, located at the left side of the detector.

### 2.1. Feasibility Study for Incoming Particle Direction Discrimination

The feasibility of the just exposed idea was verified by means of an extensive series of Monte Carlo simulations. The material chosen for the radiator was Plexiglas, with a refraction index slightly varying from 1.481 to 1.505 with increasing photons wavelength from 1.145 eV to 3.064 eV. For a value of the refraction index equal to 1.49, for photon energy of 2.066 eV, the thresholds for Cherenkov emission are $E_{th}\left(\mu \right)=142.525\mathrm{MeV},\;E_{th}\left(e^{-}/e^{+}\right)=0.689\mathrm{MeV},E_{th}\left(p\right)=1265.66\mathrm{MeV},$ for muons, electrons/positrons and protons respectively.

Different configurations of the Cherenkov detector were simulated by means of the Geant4 toolkit, changing the plate thickness and the number and arrangement of the light sensors. The light sensitive side of each radiator is instrumented with Silicon PhotoMultipliers (SiPMs) of $6\times 6\;{\mathrm{mm}}^{2}$. The best strategy to distinguish the incoming direction of the particle was established by studying the number of SiPMs that produce a signal higher than a suitable threshold on each side of the detector, which in the following will be indicated as SiPM “fired”.

The results of each set of simulations are reported as the percentage of successful recognized directions (“Successful tagged”). An event is considered well reconstructed when the discrimination condition is satisfied by only one instrumented side of the detector. The failure rate stands for the percentage of events when both instrumented sides pass the discrimination test—and it is impossible to establish which of them is the correct side—or no one. This last scenario is useful to take into account border effects, i.e., particles that enter the detector from an instrumented face and exit the second radiator from a lateral side covered by coating foil or very near to the border; the Cherenkov light cone is directed, partially or completely, toward the lateral side and there are not sufficient photons to trigger the discrimination condition. In this case the incoming direction is not recognized at all. Furthermore, it is possible that no side passes the discrimination condition for low energy muons, when the angular aperture of the Cherenkov light cone is too low to hit a sufficient number of SiPMs or when β of the particles is under threshold. The mean number of SiPMs fired for each event is also included in the results.

Four different detectors set-up were simulated, varying the size of the radiators and/or the number of SiPMs. In every case, the light sensors were placed following a regular pattern with SiPMs equally spaced. The simulated arrangements are:

1. Radiator size $30\times 240\times 240\;{\mathrm{mm}}^{3}$, 13×13 SiPMs array;
2. Radiator size $20\times 240\times 240\;{\mathrm{mm}}^{3}$, 13×13 SiPMs array;
3. Radiator size $20\times 240\times 240\;{\mathrm{mm}}^{3}$, 16×16 SiPMs array;
4. Radiator size $20\times 240\times 240\;{\mathrm{mm}}^{3}$, 20×20 SiPMs array.

**Figure 3:**
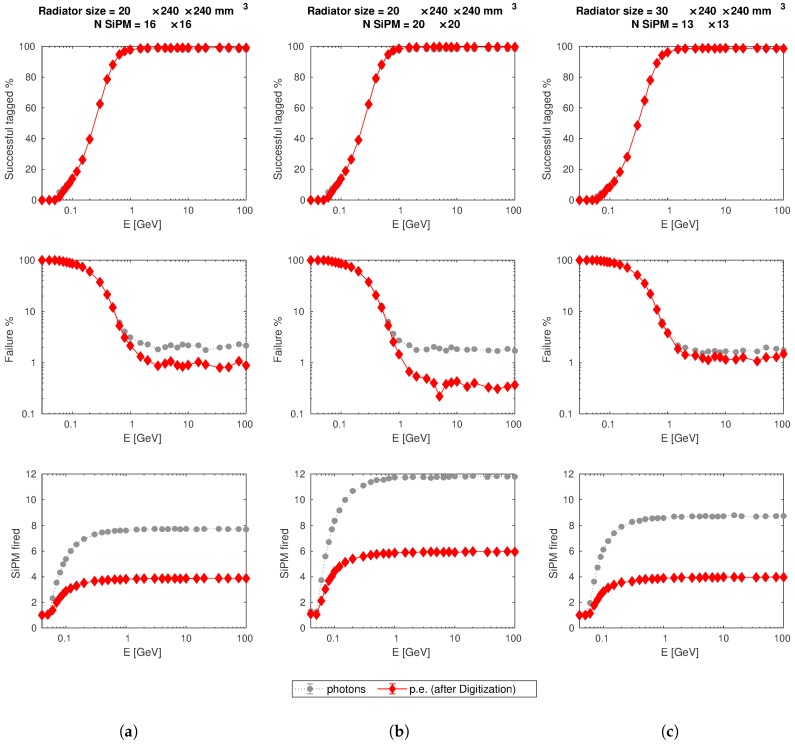
Results of Geant4 simulation as a function of the kinetic energy *E* of the primary particles (muons). Each column of subplot (**a**–**c**) refers to a different simulation scenario, in which radiator size and/or number of SiPM have changed, as shown in the title. The mean number of SiPM fired at each energy was calculated on successful reconstructed events only. The vertical error bars (equal to ±1σ) are drawn, but not visible due to the small extension.

Excluding the second set-up in which the number of SiPMs was insufficient, in the other scenarios the failure rate is lower than 2% for muons with kinetic energy greater than 1 GeV. The results for successful configurations are reported in Figure 3. These results were obtained with two primary particle sources, one for each entrance face of the detector, with the same geometric configuration. Each run of the simulation consists of $10^{4}$ muons shot randomly from one the two sources and with random direction, with angular distribution limited in order to hit the detector, as described in Section 4.3.

The analysis of the simulations output was developed in MATLAB [16] software environment, by means of which the figures that summarize the results were produced. It is possible to notice that for each subplot of Figure 3 there are two different data series, one for Cherenkov photons and the other for photo-electrons (p.e.) generated after applying the “digitization” procedure that takes into account the SiPMs properties, including dark noise, described in detail in the following.

For each scenario the best parameters for direction tagging were found and the results of Figure 3 refer to the following conditions: the threshold to consider a SiPM “fired” is equal to 3.5 photons or p.e., respectively; the number of sensors fired has to be higher than 1 (panels in Figure 3a,c), or 2 only for the case of 20×20 SiPMs (panels Figure 3b), on a single side to assign uniquely the incoming direction.

Starting from the results showed, it is possible to affirm that the extensive simulations study conducted by means of Geant4 and studied with MATLAB proves the principle of the new technique proposed for background discrimination in muography experiment. The efficiency of the set-up depends on the thickness of the radiator and on the number and arrangement of the SiPMs, but various configurations were found which give a failure rate lower than 2% at saturation energy.

It is important to stress again that the working principle of the Cherenkov-tag detector relies on the capability of light absorbing coating to stop the Cherenkov photon in it, avoiding that they can pass from the first to the second radiator traversed by the muon or that they can be reflected back to the first instrumented surface. A material with absorbance almost equal to 100% for a wide range of photon energy is not difficult to obtain. Instead, particular attention need to be devoted to the coupling between radiator and coating in order to avoid any air gap that could generate internal reflection in the radiator.

### 2.2. Position Measurement

**Figure 4:**
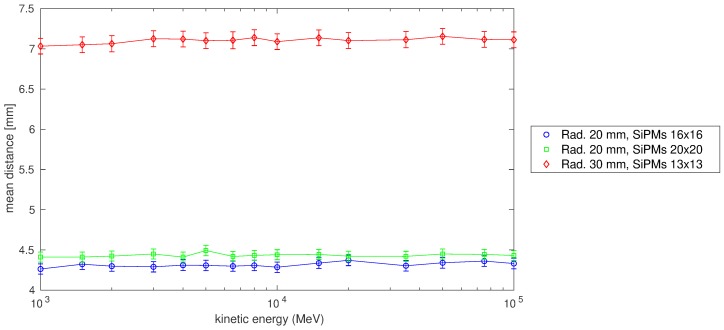
Mean distance between muon exit point from the radiator and 2D Gauss fit centroid in mm, for energies from $10^{3}\;\mathrm{MeV}$ to $10^{5}\;\mathrm{MeV}$. In the legend are reported the radiator width in mm and the number of SiPMs in the array of sensors on the opposite face of the two radiator. The vertical error bars are equal to ±3σ standard deviation of each mean distance distribution.

The possibility to use the same detector to access other information beyond the incoming direction tagging was also studied. In particular, the possibility to reconstruct the position of the crossing muon from the SIPM signals was investigated. The results of this preliminary study are reported in Figure 4. The muon position at the exit point from the radiator was reconstructed by means of a two-dimensional Gauss fit on the matrix of the number of p.e. counted by each SiPM. The mean distance between the muon exit point and the centroid obtained from fit is between 4.0 and 7.5 mm, depending on the SiPM arrangement, for muon kinetic energies at which the failure rate is lower than 2%. This result could represent an advantage respect to TOF measurement alone, because it makes accessible another position measure, with a resolution comparable with that of a detector based on 1 cm strips, for particle tracking in a detector like that of MEV project, improving its precision on trajectory reconstruction.

## 3. Discussion

The detector was initially thought as an upgrade for the muon tracker telescope already built and operating inside the MEV project. Plexiglas was chosen as radiator material to ensure high transparency to Cherenkov light, even if the UV wavelengths are cut down because its absorption length drops from several meters to only a few millimeters in this spectral region.

From the careful study of simulations was observed that Cherenkov light emission by muons was not the unique relevant physical process. In fact, with a probability that depends on primary particle energy, muons can interact with the radiator medium, generating electrons by scattering. The energy of the scattered electrons, which depends, in turn, on the energy of primary particle, is of the order of $10^{3}$ keV for the simulated muon energies. Since the energy of secondary electrons is higher than threshold for these particles, as established by Equation (1), they generate Cherenkov radiation too.

The scattering of an electron inside the radiator takes place at a random point and the direction of the electron is also random respect to that of the muon. In addition, electron path is short and this results in several Cherenkov photons emitted by the electron is negligible respect to that induced by fast muons.

Instead, for primary particle with energy lower than threshold, Cherenkov photons could be produced only by scattered electrons and they could mimic the signal of a higher energy muon leading to a wrong reconstruction of the incoming direction. This is the main reason because the percentage of successful tagged events is very near to zero for muon kinetic energy in the range of 30–50 MeV in Figure 3. Slow muons loss all or the major part of their kinetic energy into the radiator and experience a strong Multiple Coulomb Scattering (MCS) such that they are heavily deflected respect their incoming directions or completely stopped in the detector. If we consider a system with two tracking planes and a direction discrimination detector in the middle of them, slow muons will not be a problem because after traversing the Cherenkov radiator they will be scattered outside the field of view of the whole system and, without coincidence on the second tracking plane, the acquisition of the signal will not be triggered. Otherwise, if a slow muon deflects and hit anyway both tracking planes, a linear fit discrimination will reject this event because the three impact points will be not aligned. Indeed, shutting down the signal produced by muons deflected more than 1${}^{\circ }$, the efficiency is nearly zero, i.e., the discrimination of the incoming direction works as expected. The few failures are due to the digitization noise.

**Figure 5:**
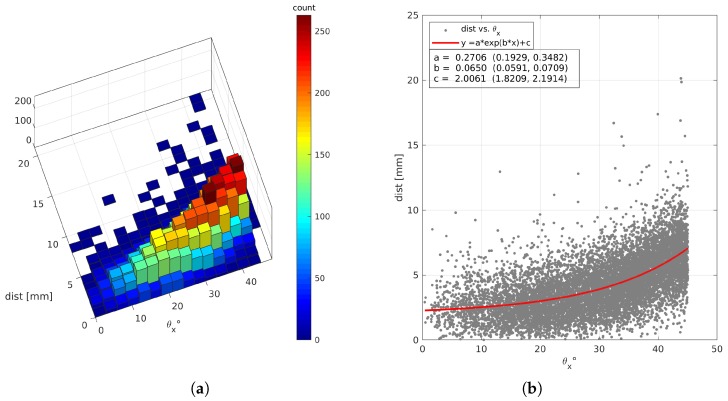
Distribution of the distance between muon exit point from the radiator and centroid of the 2D-Gaussian fit as a function of the angle $\theta_{x}$ between muon direction and the normal respect to plate. The plots refer to a scenario with radiator thickness equal to 20 mm and an array of 16×16 SiPMs; the kinetic energy of muons at the source is $10^{4}\;\mathrm{MeV}$. Plot (**a**) is a two-dimensional histogram reported to display better the counts distribution over ($\theta_{x}$, dist.) plane. Plot (**b**) includes a fit with an exponential function to suggest a possible relation between distance and $\theta_{x}$.

Regarding the position measurement, the results displayed in Figure 4 shows that for configurations with a radiator thick 20 mm and a sufficient number of SiPMs the resolution is about 4 mm, which is comparable with that of a tracking plane of MEV telescope (∼$10/\sqrt{12}\;\mathrm{mm}$). However, this numbers depends on the angular distribution of primary particle as shown in Figure 5.

To better evaluate the position measurement performance of Cherenkov-tag detector the expected angular distribution of muon trajectories should be considered, but it is possible to infer that if the major part of particle tracked have a direction close to the normal, the distance values reported in Figure 4 will be smaller. Until now, the best performance in terms of tracking resolution in muography experiment were achieved by means of gaseous detector [17,18,19] or nuclear emulsions [20,21], but with the latter the time information is inaccessible. To compare the performance of a muon tracker completely based on the new technique with an apparatus like that described in ref. [8], it is required, at least, a simulation that includes three or more tracking planes, but this is beyond the scope of this work.

If only the results of Figure 3 about the capability of direction discrimination are considered, it will be difficult to establish which of the simulated configurations is the best. In a muography experiment to investigate the internal structure of large object such a volcano, the particles with low energy can be misleading because they are strongly deflected by MCS and their trajectory could be wrongly back-projected. This consideration, together with the advantage of limited number of electronic channels for signal read-out, could lead to chose the set-up with a thicker radiator and a lower number of SiPMs. However, if the purpose of the system includes the position measurement, a closer spacing between light sensors will be useful. The configuration with two radiators, each 20 mm thick, and a regular array with 16×16 SiPMs is the best between the ones investigated, but other arrangements are unde study.

In fact, for applications outside of a common laboratory, power consumption is a key factor for the feasibility of the experiment. A Cherenkov-tag detector with 1 m^2^ of active area for the upgrade of the MEV telescope, based on a scaled version of the prototype simulated with 16×16 SiPMs for an area of 24×24 cm^2^, will have 4096 SiPMs for instrumented side. The number of power supply modules required will be equal to 128 and each one needs 10 mA of current. A typical 32-channel ASIC for SiPM readout needs about 60 mA and the detector require 128 of it. For an easy integration of the Cherenkov-tag module with the existing telescope, the readout should be managed by means of System-on-Module (SOM), manufactured by National Instruments, and the total number of channels requires 8 SOM. In conclusion, if the detector is powered by a battery with an output equal to 12 V, the power consumption of the Cherenkov detector will be equal to about 350 W and it will require a dedicated solar panel.

Next step will be the comparison between experimental results in a reduced scale prototype and simulations. A $20\times 60\times 60\;{\mathrm{mm}}^{3}$ size prototype is under construction. Many solutions for the material of the radiator and the front-end electronics will be tested. For example, the use of optical gel in place of the plexiglass, could improve the transmission for UV Cherenkov photons and the optical coupling to PS optical window. The need for the measure of the number of photons detected by each SiPM, instead of a simple threshold, imposes severe constraints in the electronic chain regarding dead time and trigger strategy.

## 4. Methods

The simulations were performed using Geant4 [22], a toolkit for simulation of the passage of particles through matter. This toolkit is not specifically developed to work as a ray-tracer, but was chosen for this task because it accomplishes the generation of optical photons according the physical process involved in the interaction between a medium and a primary particle from the source.

Geant4 provides a general model framework that allows the implementation of alternative physical models to describe the same process. To keep the simulation more general as possible and avoid to neglect some unexpected physical processes, the Physics List uses the approach of G4VModularPhysicsList. Technically speaking, a physics list can be implemented by specifying all the necessary particles and attaching to them the associated physics processes, but this requires a complete understanding of the whole physics involved. G4VModularPhysicsList, that is a sub-class of G4VUserPhysicsList, allows a user to organize physics processes into “building block”, or “modules”, and compose a physics list of such modules. This concept allows to group together desired combination of selected particles and related processes. The modules included into the physics list of the simulations discussed here are:

- G4EmStandardPhysics;
- G4DecayPhysics;
- G4EmExtraPhysics;
- G4HadronElasticPhysics;
- G4HadronPhysicsFTFP_BERT;
- G4OpticalPhysics.

A description of the available physics models and processes within the Geant4 toolkit can be found in ref. [23].

### 4.1. Detector Construction

Both radiator tiles are made of Plexiglas ($C_{5}H_{8}O_{2}$, density $=1.19g/{\mathrm{cm}}^{3}$) with sizes $240\times 240\;{\mathrm{mm}}^{2}$ along *y* and *z* coordinates and 20 or 30mm along *x* coordinate. Each tile has the lateral faces, those perpendicular to *y* and *z* axes, coated with a thin foil of polyvinyl chloride (PVC, $C_{2}H_{2}{\mathrm{Cl}}_{2}$, density $=1.7g/{\mathrm{cm}}^{3}$), 180 μm thick. One of two wider faces, perpendicular to *x* axis, is also coated with PVC foil, whereas the other one is left uncovered in order to allow the optical coupling with the light sensors. The two plates are arranged such that the larger coated surfaces face each other in contact.

Each SiPM is constructed as a mother volume whit sizes $1.45\times 6.0\times 6.0\;{\mathrm{mm}}^{3}$, along *x*, *y* and *z* coordinates respectively, made of Silicon (Si). A second thinner box is placed inside the mother volume, with sizes $0.3\times 6.0\times 6.0\;{\mathrm{mm}}^{3}$, aligned along the *x* coordinate to a surface of the Si box. This inner volume represents the photosensitive window of the SiPM and is made with epoxy resin (CHO, density $=1.0g/{\mathrm{cm}}^{3}$). The “photosensitive surface” (PS) is a metal slab at the back end of the epoxy box that is only a very rough approximation of the real thing since it only absorbs or detects the photons based on its efficiency. The SIPMs are placed with the epoxy window faced to the uncoated surface of the radiator and arranged as an equally spaced $N_{S}\times N_{S}$ square array.

The world volume, which contains all the other volumes, is filled with air.

### 4.2. Optical Properties

To correctly simulate the transportation of optical photons, Geant4 requires the user to provide the optical properties for both bulk materials and surfaces between them. This is a crucial point about processes involving optical photons, because without a documentation or a measure of the optical properties of the materials the optical physics processes will not be activated. The main parameters used in the simulations are listed in Table 1.

When a photon arrives at a medium boundary its behavior depends on the nature of the materials that compose it. Surface boundaries may be formed between two dielectric materials or between a dielectric and a metal. They can be defined in two ways: between two specific volume (G4LogicalBorderSurface) or around one volume (G4LogicalSkinSurface). The first requires that the two volume forming the surface and their optical properties have to be specified by the user. If a G4LogicalSkinSurface is attached to a volume, its properties are applied to every surface of it.

When a G4OpticalSurface is defined, the user can specify G4OpticalSurfaceModel, i.e., the reflection physics model, G4OpticalSurfaceFinish and G4OpticalSurfaceType between dielectric-dielectric and dielectric-metal. The Geant4 code allows the user to select one of the two optical reflection models, the glisur model and the unified one. The glisur model assumes that the surface is made of micro-facets, where a micro-facet is randomly selected from a distribution each time a reflection occurs. The specular reflection is calculated based on the micro-facet orientation. In the unified model four kinds of surface reflection are possible: specular spike, specular lobe, backscatter and lambertian. If no G4OpticalSurface is defined, the reflection is simulated as a geometric reflection at a perfectly smooth optical surface, i.e., applying Snell’s law. The surface finish can be set among:

- polished, smooth perfectly polished surface;
- polishedfrontpainted, polished top-layer paint;
- polishedbackpainted, polished (back) paint/foil;
- ground, rough surface;
- groundfrontpainted, rough top-layer painted;
- groundbackpainted, rough (back) paint/foil.

In the simulations, the properties for the two defined surface boundaries were kept constant. A G4LogicalBorderSurface was specified between each radiator and the PVC wrapping, with optical surface properties specified as:

- G4OpticalSurfaceModel: unified;
- G4OpticalSurfaceFinish: polishedbackpainted;
- G4OpticalSurfaceType: dielectric_dielectric.

A G4LogicalSkinSurface around the epoxy window volume was defined with the following optical surface properties:

- G4OpticalSurfaceModel: glisur;
- G4OpticalSurfaceFinish: polished;
- G4OpticalSurfaceType: dielectric_metal.

A more detailed explanation about simulation of optical physics in Geant4 can be found in ref. [24].

### 4.3. Particle Source

The particle source was implemented by means of G4GeneralParticleSource (GPS), which allows to specify spectral, spatial and angular distribution of the primary particles. As stated before, a primary particle, i.e., a muon for simulations here discussed, were shot randomly from one of the two sources defined, one for each instrumented side of the detector. Both sources had the same position distribution which consisted of a plane square with 44 mm side, parallel to detector plates and at a distance of 95 mm from the farther instrumented face. The angular distribution, that determines the directions in which the particles depart from the source point, was chosen as isotropic, specifying upper and lower limit for θ equal to 0 and 0.786 rad, respectively. Because particle direction are related to the user defined angular distribution by the following equations:

(3) $$\begin{array}{cc} P_{x} & =-\sin\theta \cos\phi \\ P_{y} & =-\sin\theta \sin\phi \\ P_{z} & =-\cos\theta \;, \end{array}$$

**Figure 6:**
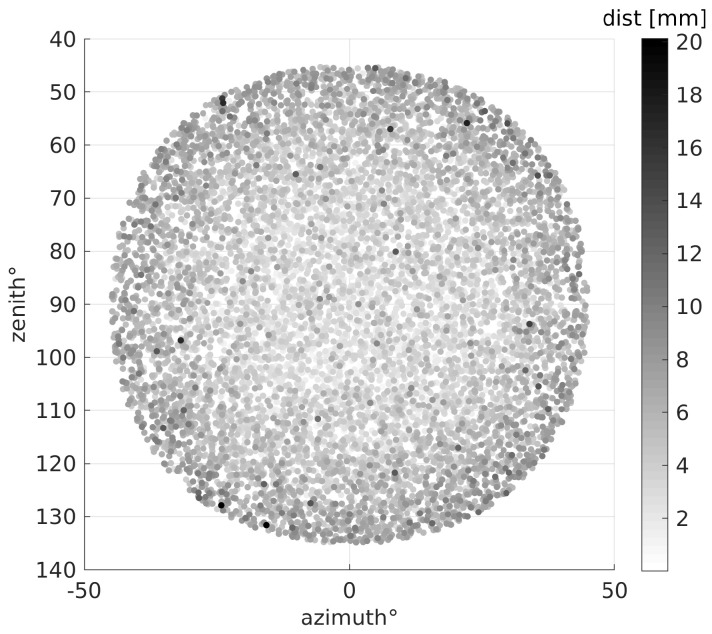
Distribution of the distance between muon exit point from the radiator and centroid of the 2D-Gaussian fit as a function of the polar angles in the reference system of the particle source. The plots refer to a scenario with radiator thickness equal to 20 mm and an array of 16×16 SiPMs; the kinetic energy of muons at the source is $10^{4}\;\mathrm{MeV}$.

θ=0 corresponds to a particle shot perpendicular to detector plates, as in the example of Figure 2. The ϕ angle was not limited. The resulting particle distribution over the exit surface of the detector is shown in Figure 6.

### 4.4. Digitization

The analysis of the simulation output was developed in MATLAB. The number of generated photons which reach the PS at the back-layer of the epoxy window is counted. This number takes into account Cherenkov radiation produced by primary muons and secondary electrons. The digitization procedure is needed to convert the number of photons, $N_{ph}$, in the given event, into the number of p.e. generated in SiPM. Because the structure of the SiPM is not simulated, a statistical approach is required for the conversion [25]. The number of p.e., $N_{pe}$, is random generated from the Poisson distribution with mean parameter $N_{ph}$ and takes into account the photon detection efficiency (PDE) and the fill factor *f* of the SiPM, as $N_{pe}=\mathrm{poissrnd}\left(N_{ph}\right)\times \mathrm{PDE}\times f$, where $\mathrm{poissrnd}\left(\lambda \right)$ is the MATLAB function to extract random number from Poisson distribution with mean parameter λ. PDE and *f* are set equal to 0.5 and 0.74 respectively, taking as reference the performance of novel SiPMs manufactured by Hamamatsu Photonics [26], which are likely candidates to be used for the first prototype of the Cherenkov-tag detector. The digitization procedure adds also a Gaussian noise to the signal, with mean and sigma equal to 1 both, in order to mimic the dark current rate of the SiPM. To consider the probability that a dark signal occurs in coincidence with a muon generated event, a parameter, pCoinc, is introduced, given by the product between muon reference rate (100 Hz), expected dark rate (10 kHz) and time coincidence window (10 ns). The digitization procedure extract a matrix, prob, of random number uniformly distributed in the interval $\left(0,1\right)$, and add the Gaussian noise only to the pixels in which pCoinc≤prob.

### 4.5. Position Measurement

When the incoming direction of the primary muons is correctly discriminated, an additional analysis can be done on the matrix of $N_{pe}$ for each SiPM of the instrumented radiator side which passes the discrimination test. It consists of a surface fit on 3D set of points. The *y* and *z* coordinates of the SiPM center positions are set as independent variables, while the corresponding $N_{pe}\left(y,z\right)$ was the dependent one. The two-dimensional fit function is a 2D rotated Gaussian:

(4) $$\begin{array}{c} N_{pe}\left(y,z\right)=\\ a+b\;\exp\left\{-{\left[\frac{\left(y-c_{1}\right)\;\cos\left(t1\right)+\left(z-c_{2}\right)\sin\left(t_{1}\right)}{w_{1}}\right]}^{2}-{\left[\frac{-\left(y-c_{1}\right)\sin\left(t_{1}\right)+\left(z-c_{2}\right)\cos\left(t_{1}\right)}{w_{2}}\right]}^{2}\right\}, \end{array}$$

with the following parameters:

- *a*, offset along $N_{pe}$ coordinate;
- *b*, amplitude of the 2D gaussian;
- $c_{1}$, centroid *y* coordinate of the 2D gaussian;
- $c_{2}$, centroid *z* coordinate of the 2D gaussian;
- $t_{1}$, angle of rotation for the 2D gaussian;
- $w_{1}$, width along *y* of the 2D gaussian;
- $w_{2}$, width along *z* of the 2D gaussian.

The reconstructed position of a muon going out the last radiator along its path is assigned equal to $\left(c_{1},c_{2}\right)$ on the (y,z) plane at *x* equal to the border of the radiator. Then the distance between $\left(c_{1},c_{2}\right)$ and the true exit position of the muon, retrieved from the simulation output is calculated to investigate the precision of this measurement, as shown in Figure 4.

## 5. Conclusions

In conclusion, it is possible to affirm that the feasibility study conducted and exposed in this paper opens a new promising possibility to reduce the background noise in muography applications. The working principle of the Cherenkov tag detector works well in simulations. Even if other aspects could be investigated, the next fundamental steps will be a comparison and a fine tuning between simulations and the experimental study of a prototype, which include many technical constrains not reproducible in simulations.

## Figures and Tables

**Table 1:** Materials and surfaces optical properties implemented in the simulations. When two values are specified, the corresponding optical property varies with photon energies that spans from 2.00 to 4.136eV.

Optical Properties	
Refractive index of Air	1.00
Refractive index of Plexiglass	1.481–1.505
Absorption length of Plexiglass	5.40 m–1 mm
Refractive index of Epoxy	1.55
Absorption length of Epoxy	4.20 m–1 mm
Efficiency of PS	1.00
Reflectivity of PS	0.05
Reflectivity of PVC	0.01
